# Comparative Study of Novel Noninvasive Cerebral Autoregulation Volumetric Reactivity Indices Reflected by Ultrasonic Speed and Attenuation as Dynamic Measurements in the Human Brain

**DOI:** 10.3390/brainsci10040205

**Published:** 2020-04-01

**Authors:** Basant K. Bajpai, Rolandas Zakelis, Mantas Deimantavicius, Daiva Imbrasiene

**Affiliations:** 1 Health Telematics Science Institute, Kaunas University of Technology, LT-51423 Kaunas, Lithuania; 2 Department of Health Promotion and Rehabilitation, Lithuanian Sports University, LT-44221 Kaunas, Lithuania; 3 Institute of Physiology and Pharmacology, Lithuanian University of Health Sciences, LT-44307 Kaunas, Lithuania

**Keywords:** ABP = arterial blood pressure, CPP = cerebral perfusion pressure, CA = cerebrovascular autoregulation, ICP = intracranial pressure, PRx = pressure reactivity index, CBF = cerebral blood flow, VRx = volumetric reactivity index, TOF = time-of-flight

## Abstract

This is a comparative study of two novel noninvasive cerebrovascular autoregulation (CA) monitoring methods based on intracranial blood volume (IBV) changes in the human brain. We investigated the clinical applicability of the new volumetric reactivity index (VRx2), reflected by intracranial ultrasonic attenuation dynamics for noninvasive CA monitoring. The CA was determined noninvasively on 43 healthy participants by calculating the volumetric reactivity index (VRx1 from time-of-flight of ultrasound, VRx2 from attenuation of ultrasound). The VRx was calculated as a moving correlation coefficient between the arterial blood pressure and noninvasively measured IBV slow waves. Linear regression between VRx1 and VRx2 (averaged per participants) showed a significant correlation (*r* = 0.731, *p* < 0.0001, 95% confidence interval [0.501–0.895]) in data filtered by bandpass filtering. On the other hand, FIR filtering demonstrated a slightly better correlation (*r* = 0.769, *p* < 0.0001, 95% confidence interval [0.611–0.909]). The standard deviation of the difference by bandpass filtering was 0.1647 and bias −0.3444; and by FIR filtering 0.1382 and bias −0.3669. This comparative study showed a significant coincidence of the VRx2 index compared to that of VRx1. Hence, VRx2 could be used as an alternative, cost-effective noninvasive cerebrovascular autoregulation index in the same way as VRx1 values are used.

## 1. Introduction

The mechanisms of cerebral autoregulation remain poorly understood, especially in humans. Cerebrovascular autoregulation refers to the brain’s ability to maintain constant cerebral blood flow (CBF) with changes in cerebral perfusion pressure (CPP) [1] based on cerebral metabolism independent of fluctuations in systemic arterial blood pressure (ABP). This process is controlled by multifactor mechanisms, including myogenic, metabolic, and neurogenic metabolic mechanisms [2,3,4].

Cerebral autoregulation (CA) is the primary factor that influences treatment outcomes in brain trauma patients [5,6]. When CA is impaired, the outcomes for traumatic brain injury (TBI) patients are significantly impacted. Autoregulation has been described as a balancing act between vasoconstriction and vasodilation because the resistance of the cerebrovascular bed accepts slow dynamic changes in cerebral perfusion pressure. The impairment of CA has the most significant influence on these outcomes, which means that it is essential to explore CA continuously over time [7,8,9,10].

Several existing methods are used to estimate CA status based on measuring fluctuations in CPP and CBF along with cerebral vascular resistance changes in CPP (or ABP) [11,12,13,14]. Generally, existing CA assessment methods are based on the application of the surrogate physiological parameters that makes it practically impossible to replace them with noninvasive CBF monitoring. The objective of clinically invasive methods for assessing continuous CA is to determine the pressure reactivity index (PRx) by the moving Pearson’s correlation coefficient (*r*) between slow waves of ABP(*t*) and ICP(*t*) over a time window of a few minutes. With intact CA, slow increases in ABP cause vasoconstriction, which is followed by a decrease in ICP, resulting in a negative PRx; however, while CA is impaired, a rise in luminal ABP can lead to passive cerebrovascular dilation and an increase in cerebral blood volume and ICP. In such cases, PRx will be positive [15,16,17,18,19]. It has been found that PRx values above a critical level associated with brain vascular deterioration can cause death [20,21]. The crucial threshold for PRx was suggested and recommended by various scientists (i.e., values above 0.2 or 0.25 for PRx being associated with impaired status and those close to zero or negative being associated with intact autoregulation) [22,23,24].

PRx is one of the widely used indices for CA monitoring [15,16,17,18,19,20,21,22,23,24], as no gold standard exists. However, an important limitation of this approach is that it is invasive (i.e., ICP sensors must be inserted into brain ventricles or parenchyma tissue).

All other existing noninvasive methods attempt to find a surrogate parameter that could replace CBF monitoring so that the related CA index can be calculated [25]. Among several noninvasive methods, the transcranial Doppler (TCD) method for assessing CA noninvasively is primarily used. The middle cerebral artery (MCA) for blood velocity is used instead of CBF to estimate the TCD, depending on the autoregulation index (Mx) as a moving correlation coefficient between the MCA and ABP. Similarly, other methods, such as near-infrared spectroscopy (NIRS), are used to estimate the correlation coefficient between the measured local oxygen saturation and ABP. These are referred to as the cerebral oximetry (Cox or Tox) index [26,27] and the hemoglobin volume index (HVx), which are monitored with a moving linear correlation of blood pressure to cerebral blood volume near-infrared spectroscopy [28]. The local dependency is a limitation of these noninvasive methods [25].

A novel noninvasive technology (Certification (CE) marked device) was developed by the Health Telematics Science Institute at the Kaunas University of Technology (Kaunas, Lithuania). ICP*(t)* slow waves were replaced by IBV*(t)* slow waves to estimate CA status, which was included in the calculation of the noninvasive volumetric reactivity index VRx*(t)* as a moving correlation coefficient between the slow IBV*(t)* and ABP*(t)* waves [25]. Clinical applicability of the VRx has been proven by several clinical studies conducted by the Health Telematics Science Institute at the Kaunas University of Technology (Kaunas, Lithuania), such as the noninvasive ultrasonic VRx based on time-of-flight measurements with the invasive PRx on 61 patients with brain injuries and showed excellent coincidence to reflect autoregulation [25]. Another comparative study with 11 patients with brain injuries revealed a significant relationship associated with VRx and PRx outcomes [29], which indicated that VRx reflects autoregulation the same as PRx. Additionally, the applicability of the noninvasive IBV monitoring technique for cerebrovascular autoregulation monitoring has reflected the similarity between invasive ICP and noninvasive IBV [29,30,31], which are used to derive the reactivity indices (PRx, VRx). In this study, we investigated novel noninvasive ultrasonic methods developed by the Health Telematics Science Institute at the Kaunas University of Technology (Kaunas, Lithuania) based on real-time monitoring of ultrasound speed and compared that with attenuation dynamics. We aimed to determine similarities in both the time-of-flight and attenuation channel in order to use VRx2 (attenuation based index) as an alternative technology. This is closely similar to the time-of-flight technology related to the acoustic path, and this path crosses arterioles (small vessels), responsible for autoregulation, while attenuation shows this integrated reaction of small vessels to changes in blood pressure.

The clinical applicability of novel noninvasive ultrasound attenuation based VRx2 is comparable with time-of-flight based VRx1, which is already in use for clinical studies. The attenuation ultrasound based VRx2 is an attractive, cost-effective alternative to the time-of-flight based VRx1 and even less expensive than TCD (transcranial Doppler) and potentially easy for hardware miniaturization. A comparative study was performed by determining VRx1 and VRx2 calculations.

## 2. Materials and Methods 

The methods were performed according to the approved guidelines. The Kaunas regional ethics committee approved this study, Approval No. BE-2-49 (16 November 2017), Kaunas, Lithuania. The data from 43 healthy participants were collected continuously by an ABP monitor (Finapres Nova) that displayed the ABP signal. Among 43 healthy participants, 33 of them were male, and 10 were female in the age range from 18 to 36 years. The criteria for the data selection for the study was high-resolution raw data.

Furthermore, for IBV monitoring, a novel noninvasive ultrasonic monitor was developed by the Health Telematics Science Institute at the Kaunas University of Technology (Kaunas, Lithuania). Further data recording and processing were accomplished by using ICM+ software (version 3, Cambridge Enterprises Ltd., Cambridge, U.K.), where the real-time calculation of the noninvasive CA indices from ABP and IBV was obtained. All the recorded data were used for post-hoc analysis to collect additional information and analyze real-time data over time.

### 2.1. Cerebral Autoregulation Assessment

CA status was monitored using a novel noninvasive ultrasonic monitor developed by the Health Telematics Science Institute at the Kaunas University of Technology (Kaunas, Lithuania) for real-time monitoring of ultrasound speed and attenuation dynamics in the human brain. The technology provides real-time information regarding IBV changes and waves in the cerebral vessels responsible for CA [29,30,31]. Such changes and waves are causes of intracranial pressure (ICP*(t)*) changes and waves [32]. The idea of the novel technology was based on the possibility of measuring intracranial blood volume changes and waves inside an acoustic path that crosses the human head, applying an ultrasonic time-of-flight method. The speed and attenuation of ultrasound reflect the density of blood, brain tissue, and cerebrospinal fluid volumes inside an acoustic path. According to the database of the IT’IS (Information Technologies in Society) Foundation (Switzerland, 2019) [33], the values of ultrasound speed (± standard deviation) in blood, brain tissue, and cerebrospinal fluid were 1578.2 (± 11.3) m/s, 1546.3 (± 20.2) m/s, and 1505.5 (± 3.5) m/s, respectively. Ultrasonic time-of-flight technology measures the time of short ultrasound pulses transmitted through the human head with picosecond resolution and the attenuation of such pulses. Both measurement results reflect the changes of blood and cerebrospinal fluid volume inside the acoustic path in different ways, which are reflected by intracranial ultrasound speed and attenuation changes caused by cardiac pulsation, respiration processes, cerebrospinal blood flow autoregulation processes, and other intracranial dynamic phenomena.

The usual invasive CA monitoring is based on ABP*(t)* and the ICP*(t)* slow wave correlation index PRx*(t)* calculation [15,34,35,36,37]. In new noninvasive technology, ICP*(t)* slow waves, was replaced by IBV*(t)* slow waves, and CA status was estimated by calculating the noninvasive VRx*(t)* as a moving correlation coefficient between the slow waves of IBV*(t)* and ABP*(t)*. Slow ABP*(t)* and IBV*(t)* B waves with a period of 0.5–2.0 min reflected the vasogenic activity of cerebrovascular autoregulation and were used for VRx*(t)* calculations [29,30,31,38]. A head frame including a pair of ultrasonic transducers on either side of the head was positioned so that the acoustic path crossed intracranial media, including parenchyma and brain ventricles, but avoiding large arteries and veins. IBV*(t)* changes and slow waves caused by vasodilatation and vasoconstriction mechanisms reflected the changes in the diameter of the parenchymal vessels responsible for maintaining a relatively stable CBF [28,31]. Vasoconstriction of arterioles and capillary vessels was the physiological autoregulatory reaction to increased mean arterial blood pressure (MAP). The blood volume inside the acoustic path decreased in cases, while that monitored by the speed of ultrasound also decreased [30,39]. The capability to sense overall integrated volumetric reactions of the brain and increased temporal resolution of CA monitoring were the primary advantages of this ultrasonic method compared with other methods based on local blood volume/velocity measurements by using near-infrared spectroscopy (NIRS) and Doppler technology applications [40,41,42,43,44,45].

The CA status of healthy participants was continuously assessed for 15 min by monitoring the time dependence of the two noninvasively recorded VRx*(t)* indices, including VRx*(t)*1, which reflected ultrasound speed dynamics, and VRx*(t)*2, which reflected ultrasound attenuation dynamics. The ABP*(t)* slow wave reference signal was taken from a noninvasive ABP*(t)* Finapres Nova monitor. Negative values of both VRx*(t)* < 0 corresponded to intact CA status, and positive VRx*(t)* values > 0 indicated CA impairment [15,28,29,33,34,35,36,37]. Two-minute moving averages of both VRx*(t)* were used to obtain a temporal resolution of CA impairments that were at least two times better than those obtained with NIRS or Doppler CA monitors. During CPB, continuous slow MAP*(t)* waves with a stable period of T = 60 s were generated by periodic (period of 60 s) 20 second breath-holds. This type of periodic modulation of O_2_/CO_2_ saturation in cerebral blood created reference MAP*(t)* slow waves and the speed of ultrasound and ultrasound attenuation slow waves as cerebrovascular blood flow autoregulatory reactions (two informative signals for two VRx*(t)* index calculations).

### 2.2. Post Hoc Analysis 

The sampling frequency employed to generate raw ABP and ICP data was 50 Hz. The recorded data were decimated to a 1 Hz sampling frequency, and bandpass filtering with a bandwidth from 0.008–10 Hz was used to extract the slow waves of ABP*(t)* and IBV*(t)* (of the attenuation and time-of-flight channels). The correlation coefficient was estimated between the bandpass filtered spectra of both channels’ slow waves.

On the other hand, the FIR (Parks–McClellan) filter was also used for a 1 Hz sampling frequency with a passband ripple of 0.1 dB to extract slow waves of ABP*(t)* and IBV*(t)* (for attenuation and time-of-flight channels). Moreover, the correlation coefficients were estimated between the FIR filtered spectra of both channels’ slow waves.

After slow wave correlation studies, noninvasive volumetric reactivity indices (VRx1 and VRx2) were calculated as a moving correlation coefficient between the slow waves of IBV*(t)* and ABP*(t)* waves from the time-of-flight and attenuation channels. Slow ABP*(t)* and IBV*(t)* B waves were obtained with a period of 1–2 min. Two separate sets of volumetric indices (VRx1 and VRx2) were estimated, one set from the bandpass filter and the other set from the FIR filter. The volumetric indices were then compared to determine the differences in both channels’ CA outcomes with different filtration approaches.

Additionally, before slow wave filtering, hyperventilation and breath-holding tests were performed for vasoconstriction and vasodilation dynamics for a few seconds to determine if both channels reacted to the physiology in the same way, when arterial blood pressure (ABP) was the same. Furthermore, pulse waves for the 10 s window were also extracted from both the time-of-flight and attenuation channels for comparison purposes.

### 2.3. Statistical Analysis

Statistical analysis was performed using the SPSS (IBM Inc., New York, NY, USA) Version 20 software package. A linear regression analysis of VRx1 and VRx2 was performed. VRx1 and VRx2 data were averaged during each simultaneous monitoring session to produce one value per participant (43 data points). Pearson’s correlation coefficient between VRx1 and VRx2 averaged data was calculated with a 3 minute time window used for each monitoring session.

## 3. Results

**Figure 1:**
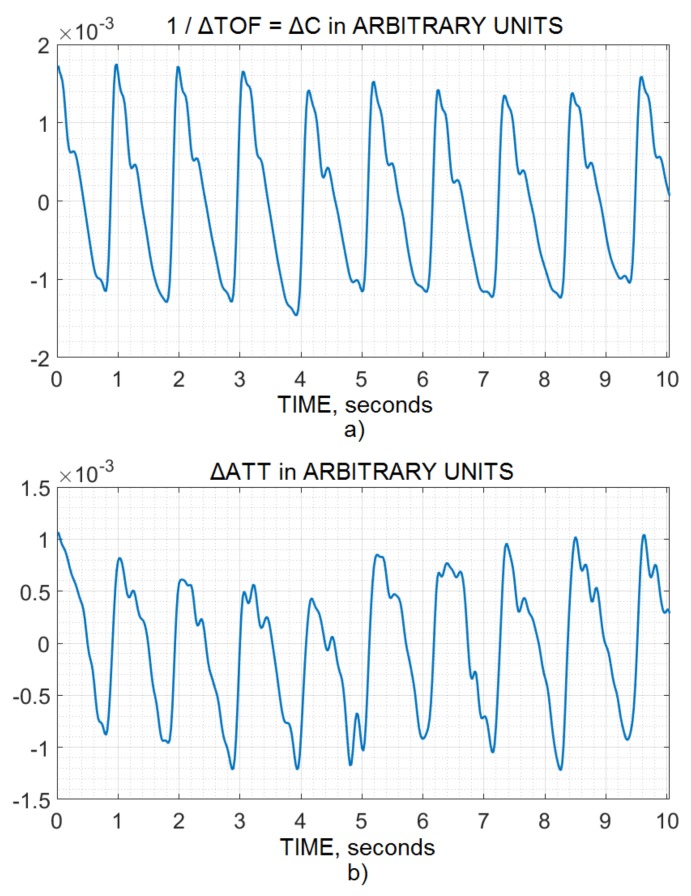
Intracranial volumetric pulse waves (causes of ICP waves) noninvasively and simultaneously recorded by (**a**) an ultrasonic time-of-flight recording method and (**b**) an attenuation recording method inside an acoustic path, which is crossing the skull and brain.

**Figure 2:**
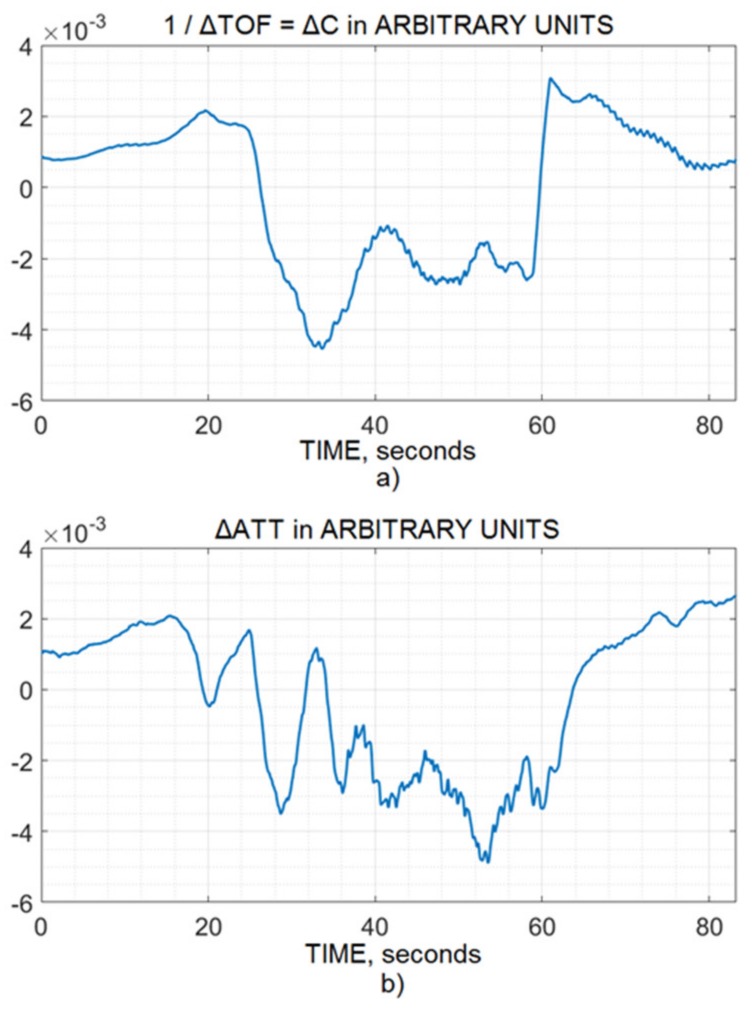
Vasoconstriction dynamics of intracranial blood vessels caused by hyperventilation test and recorded by (**a**) the time-of-flight and (**b**) attenuation channels of the ultrasonic noninvasive CA monitor.

**Figure 3:**
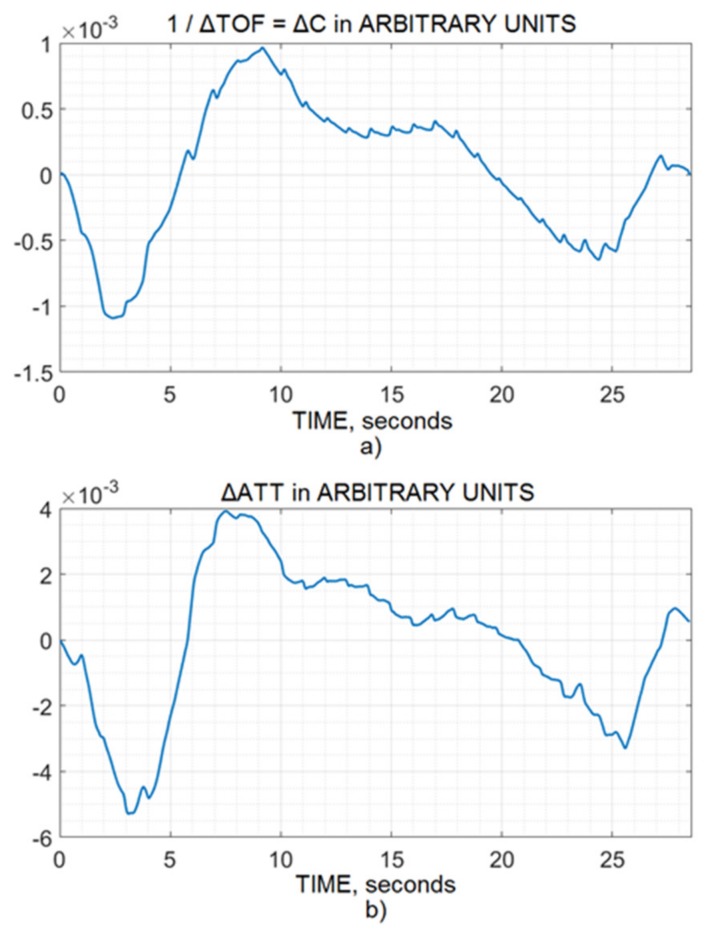
Vasodilatation dynamics of intracranial blood vessels caused by the breath-holding test and recorded by the (**a**) time-of-flight and (**b**) attenuation channels of the ultrasonic noninvasive CA monitor.

Before slow wave filtering, the pulse wave for the 10 second window was extracted from both the time-of-flight and attenuation channels, which indicated that both channels had a similar reaction to the physiology, as shown in Figure 1a,b. Furthermore, hyperventilation and breath-holding tests were performed for vasoconstriction and vasodilation dynamics for a few seconds by time-of-flight and attenuation ultrasonic noninvasive CA monitoring (see Figure 2 and Figure 3). They also reflected that both the channels reacted to the physiology in the same way when ABP was the same.

The noninvasive VRx1, which reflected ultrasound speed dynamics, and VRx2, which reflected ultrasound attenuation dynamics, indices were recorded for 43 healthy participants. The monitoring sessions were performed for 15 min for each participant. Correlation between slow waves extracted from the time-of-flight and attenuation channels were as shown in Table 1.

In Table 1, participants were grouped according to correlation outcomes between the time-of-flight and attenuation channels, where correlation from the FIR filtering approach had 33 participants with a correlation of more than 0.5 (higher correlation) and ten participants with less than 0.5 (low) correlation.

**Figure 4:**
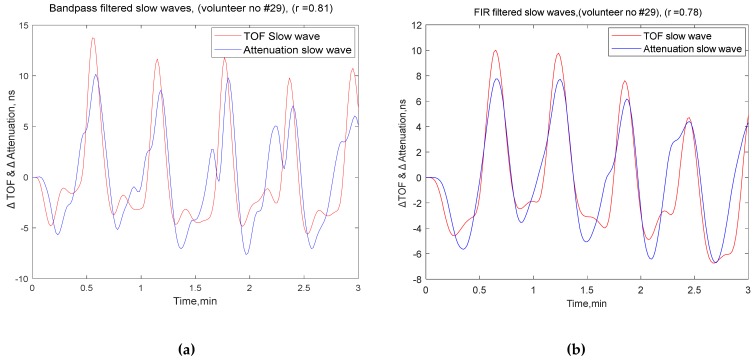
Examples of three minute periods of slow waves extracted from time-of-flight and attenuation dynamics; comparison of participants with (**a**) excellent correlation by the bandpass filter and (**b**) excellent correlation by the FIR filter.

**Figure 5:**
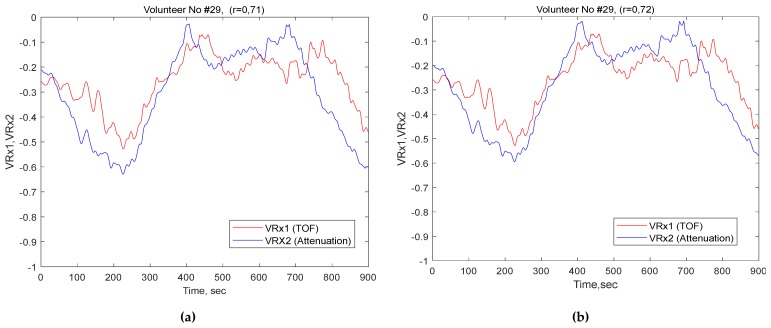
Comparison window for volumetric reactivity indices (VRx 1 and VRx2) for15 minute time with (**a**) an excellent correlation by the bandpass filter and (**b**) excellent correlation by the FIR filter.

By contrast, bandpass filtering found 31 participants with a correlation of more than 0.5 (higher correlation) and 12 participants with less than 0.5 (low), which reflected a significant correlation in the ultrasound speed dynamics’ (TOF) slow waves and ultrasound attenuation’s slow waves. Examples of comparisons between both channels’ slow waves with correlations for a three minute window are shown in Figure 4. Furthermore, examples of comparisons between VRx1 and VRx2 over 15-minute monitoring periods are shown in Figure 5.

**Figure 6:**
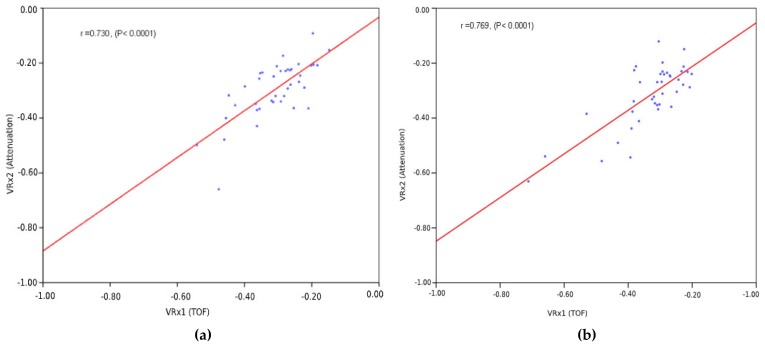
Comparison of VRx1 and VRx2 indices by regression analysis. (**a**) Bandpass filtering. The correlation coefficient between VRx1 and VRx2 indices is denoted as *r* = 0.730. The statistical significance is *p* < 0.0001. (**b**) FIR filtering. The correlation coefficient between VRx1 and VRx2 indices is *r* = 0.769. The statistical significance is *p* < 0.0001.

Three minute time-averaged (per participant in 43 pairs) VRx1 and VRx2 indices were used for linear regression, where the correlation coefficient between VRx1 and VRx2 averaged 0.731, with a 95% confidence interval of 0.501–0.895 and a statistical significance of less than 0.0001 in the studied population. Similarly, in FIR filtered data, the correlation coefficient between VRx1 and VRx2 averaged 0.769, with a 95% confidence interval of 0.611–0.909 and a statistical significance of less than 0.0001 in the studied population (see Figure 6a,b).

The linear regression plots between the pairs of VRx1 and VRx2 indices were averaged per participant’s monitoring sessions, as shown in Figure 6a,b. There were significant statistical similarities between both the ultrasound speed dynamics (time-of-flight), ultrasound attenuation dynamics, and their CA indices (VRx1 and VRx2), while the FIR filtered approach exhibited slightly better correlation outcomes compared to bandpass filtration.

**Figure 7:**
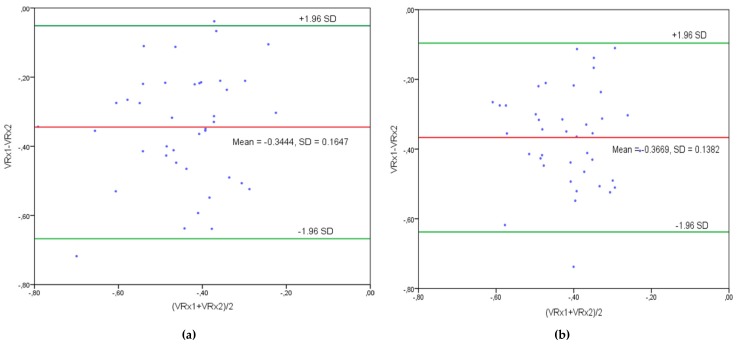
Comparison of VRx1 and VRx2 indices by Bland–Altman plots. (**a**) Bandpass filtering. The standard deviation of the difference between the indices is SD 0.1647. Bias is −0.3444. (**b**) FIR filtering. The standard deviation of the difference between the indices is SD 0.1382. Bias is −0.03669.

The standard deviation of the difference between VRx1 and VRx2 was 0.1647, and the bias between VRx1 and VRx2 was −0.3444 by bandpass filtering; while in FIR filtered data, the standard deviation of the difference between VRx1 and VRx2 was 0.1382, and the bias between VRx1 and VRx2 was −0.3669 (see Figure 7a,b). Bland–Altman plots between the pairs of VRx1 and VRx2 indices averaged per participant’s monitoring sessions are shown in Figure 7a,b. All these outcomes reflected that there was good agreement between both the ultrasound speed dynamics (TOF) and ultrasound attenuation dynamics based indices (VRx1, VRx2).

## 4. Discussion

CA status analysis was conducted for healthy participants by monitoring the time dependence of noninvasively recording the two VRx*(t)* indices, including VRx*(t)*1, which reflected ultrasound speed dynamics, and VRx*(t)*2, which reflected ultrasound attenuation dynamics. As there was no gold standard for noninvasive CA monitoring, VRx (ultrasound speed dynamics), time-of-flight was chosen as a reference index, which is CE marked and already being used for clinical studies.

Changes in IBV values are known to consist of slow respiratory and pulse waves and are associated with ICP changes [25]. Hence, the similarity among the channel’s pulse wave, vasoconstriction, and vasodilation was evidence of similarity and served as the basic foundation of this study. Figure 1, Figure 2 and Figure 3 indicate that both channels (time-of-flight and attenuation) reacted to the physiology in the same way, keeping in mind the artifacts, disturbance, and patient’s movement, which were filtered by the bandpass and excellent FIR filtration methods.

However, there were some differences between the channels. For example, if the surplus of oxygen provoked IBV change as a result of hyperventilation, there was too much oxygen in the blood, and this caused vasoconstriction. Hence, the volume decreased in both the channels, though the difference was small. This slight difference showed that the attenuation channel was slightly different (Figure 2), which may be due to many factors, such as differences in transmission or differences in the measurement of attenuation and time-of-flight channels because a different parameter of dynamic media was measured. Generally, if blood volume increased, both channels indicated an increment, and if blood volume went down, both channels should indicate a fall.

We created a classifier; the classifier had two states: impaired (autoregulation reactivity index zero to one) and intact (autoregulation reactivity index below −1 to zero). We took an existing classifier, (VRx1) time-of-flight, clinically tested [25,29,30,31], as a reference to create another inexpensive classifier, attenuation-based reactivity index (VRx2). We compared both classifiers to know if they provided the same diagnostic information. We used linear regression and Bland–Altman methods to compare the agreement between these two methods. Linear regression showed significant correlation of 0.731, *p* < 0.0001, 95% confidence interval [0.501–0.895], in the bandpass filter. FIR filtering had a slightly better correlation of 0.769, *p* < 0.000, 95% confidence interval [0.611–0.909].

On the other hand, Bland–Altman’s direct comparison proved that both classifiers gave intact cerebral autoregulation values, as both the curves of VRx1 and VRx2 had outcomes from −1 to zero (see Figure 7a,b), which was intact autoregulation [15,28,29,33,34,35,36,37]. As we already knew that we were using healthy subjects for the study, that guaranteed an intact outcome.

The average of both VRx*(t)* values was used to achieve a temporal resolution of CA impairments’ detection that was at least two times better than that of NIRS or Doppler CA monitoring. The capability to sense overall integrated volumetric reactions of the brain and increased temporal resolution of CA monitoring were the primary advantages of this ultrasonic method compared with other methods based on local blood volume/velocity measurements using near-infrared spectroscopy (NIRS) and Doppler applications [40,41,42,43,44,45].

Several studies have been conducted that compared invasive CA indices, though most of them were compared against the invasive PRx. However, this study provided a comparison of two noninvasive volumetric reactivity indices to show that the novel attenuation-based volumetric reactivity index (VRx2) could be used as an alternative to time-of-flight based volumetric reactivity index (VRx1), where VRx1 (based on time-of-flight) is already tested in (dynamic conditions) traumatic brain injury patients, against PRx, and it is already in use for clinical studies. Time-of-flight studies [25,29] indicated that the VRx1 (tof channel) output signal highly correlated with invasive PRx*(t)*, which could be used for comparison with the attenuation channel’s signal in healthy participant studies. However, it would be recommended in the future to test the attenuation channel directly in traumatic brain injury patients against PRx. Time-of-flight is very expensive, while attenuation is very simple (measuring the amplitude of the ultrasonic pulse, which passes through the brain), cost-effective, even less expensive than transcranial Doppler, and easy to measure. Hence this technology could be instrumental in developing (or low income) countries instead of expensive time-of-flight (VRx1) measurement.

## 5. Conclusions

This comparative study of the noninvasive ultrasonic volumetric reactivity indices VRx1 (time-of-flight) and VRx2 (attenuation) monitoring was based on ultrasonic time-of-flight and reflected ultrasound attenuation measurements of IBV dynamics, which showed a significant correlation. VRx2 (attenuation) could be used as a noninvasive cerebrovascular autoregulation index in the same way as VRx1 and could be used to reflect essential information related to CA status. Compared with a bandpass filter, the FIR filter had slightly better correlation outcomes among both indices.

## 6. Limitations of the study 

This study was conducted on a small population of participants (only 43 participants). A validation study with a much larger population of healthy participants is necessary. Furthermore, there is no gold standard for the cerebral autoregulation monitoring method. Another essential factor in producing more concrete results is the comparison of volumetric reactivity index (VRx1 and VRx2) outcomes with various filtering approaches and various methods. It would be recommended in the future to test the attenuation channel directly in traumatic brain injury patients against PRx.

## Figures and Tables

**Table 1:** Correlation outcome between slow waves of TOF and attenuation channels.

Correlation Outcome (r)	Band-Pass Filtered Slow Wave	FIR Filtered Slow Wave
>0.5	31 participants	33 participants
<0.5 to 0	12 participants	10 participants
